# A Pragmatic Two-Step Screening Algorithm for Sarcopenia and Frailty in Community-Dwelling Older Adults: A Cross-Sectional Population-Based Study

**DOI:** 10.3390/life16010106

**Published:** 2026-01-12

**Authors:** Silvana Mirella Aliberti, Antonio Menini, Anna Maria Sacco, Veronica Romano, Aldo Di Martino, Vittoria Acampora, Gemma Izzo, Chiara Sorrentino, Daria Nurzynska, Franca Di Meglio, Clotilde Castaldo

**Affiliations:** 1 Department of Medicine, Surgery and Dentistry “Scuola Medica Salernitana”, University of Salerno, 84081 Baronissi, SA, Italy; dnurzynska@unisa.it; 2 Department of Public Health, University of Naples “Federico II”, 80131 Naples, NA, Italy; antonio.menini@unina.it (A.M.); annamaria.sacco@unina.it (A.M.S.); veronica.romano@unina.it (V.R.); aldo.dimartino@unina.it (A.D.M.); vittoria.acampora@unina.it (V.A.); gem.izzo@studenti.unina.it (G.I.); chiara.sorrentino4@studenti.unina.it (C.S.); clotilde.castaldo@unina.it (C.C.)

**Keywords:** sarcopenia, frailty, screening, older adults, public health, muscle function, EWGSOP2, prevention

## Abstract

Sarcopenia and physical frailty are interconnected geriatric syndromes that frequently coexist in older adults, sharing common pathophysiological pathways. However, their early detection in community settings is limited by resource constraints and by the lack of simplified, scalable diagnostic tools. This cross-sectional study aimed to estimate the prevalence and overlap of sarcopenia and frailty in a real-world public health screening programme and to evaluate the diagnostic performance of a pragmatic two-step algorithm. In September 2025, a total of 256 consecutive community-dwelling adults aged ≥65 years underwent standardized assessment using the SARC-F questionnaire, handgrip strength dynamometry, and selective bioelectrical impedance analysis (BIA). Sarcopenia was defined according to 2019 EWGSOP2 criteria, and frailty according to the Fried phenotype. Confirmed sarcopenia was identified in 37 participants (14.5%, 95% CI 10.7–19.1%) and frailty in 31 (12.1%, 95% CI 8.6–16.7%), with substantial overlap (77.4% of frail individuals also had sarcopenia; Cohen’s κ = 0.62). The two-step algorithm (Step 1: SARC-F ≥ 4; Step 2: handgrip strength and BIA only in screen-positive participants) demonstrated excellent accuracy for confirmed sarcopenia (AUC 0.913, 95% CI 0.871–0.955), with sensitivity 91.9%, specificity 81.3%, and a 53.9% reduction in BIA use. Factors independently associated with confirmed sarcopenia included older age, BMI < 22 kg/m^2^, physical inactivity, and higher SARC-F score. A simple, function-centered two-step approach enables efficient and scalable identification of sarcopenia and frailty in community settings, supporting early preventive strategies to preserve physical function.

## 1. Introduction

Sarcopenia and frailty are increasingly recognized as key determinants of adverse health trajectories in aging populations [1]. Sarcopenia, defined as the progressive and generalized loss of skeletal muscle strength and mass, represents a central component of physical decline and is associated with functional impairment, falls, disability, healthcare utilization, and mortality [2]. According to the revised European Working Group on Sarcopenia in Older People (EWGSOP2) criteria, low muscle strength constitutes the primary indicator of sarcopenia, with confirmation requiring reduced muscle quantity and severity determined through impaired physical performance [3]. Beyond its clinical impact, sarcopenia has wide-ranging socioeconomic consequences, given the rapid demographic shift toward older populations and the projected rise in dependency ratios [4].

Frailty, traditionally conceptualized through the physical phenotype proposed by Fried and colleagues, is a multidimensional syndrome reflecting decreased physiological reserve across multiple systems [5,6]. Unlike sarcopenia, which focuses on musculoskeletal health, frailty encompasses a broader vulnerability to stressors, manifesting through exhaustion, slowed mobility, low activity levels, and unintended weight loss. Although sarcopenia and frailty frequently overlap, they are distinct constructs with different etiologies, trajectories, and implications for intervention. Their intersection, however, likely identifies individuals at particularly high risk of adverse outcomes [4]. Despite extensive research, no universal gold standard exists for assessing frailty, and operational definitions vary widely across clinical and research settings. More than 20 frailty instruments have been proposed—ranging from the Frailty Phenotype to the Frailty Index, Clinical Frailty Scale, PRISMA-7, FRAIL scale, Gérontopôle Frailty Screening Tool, and electronic frailty algorithms—each emphasizing different theoretical frameworks and dimensions. This heterogeneity has hindered comparability across studies and has shown the integration of frailty screening into routine care. The FRAILTOOLS project, a large multicenter European initiative, has highlighted these inconsistencies and underscored the need for validated, setting-specific tools capable of predicting clinically relevant outcomes across acute, outpatient, primary care, and institutionalized populations [7]. Furthermore, recent efforts to develop multidimensional frailty instruments, such as the Korean Frailty Scale (KFS), which incorporates physical, psychological, and social components, confirm the growing consensus that frailty must be captured as a complex, multi-domain phenomenon and demonstrate the potential of brief, holistic tools to predict falls, disability, hospitalization, and mortality [8].

In contrast to frailty, definitions of sarcopenia have become more standardized due to EWGSOP2 and the Asian Working Group for Sarcopenia framework. However, real-world application of these criteria remains limited. Sarcopenia is increasingly understood as the result of multifactorial and interdependent mechanisms, including aging-related anabolic resistance, chronic inflammation, physical inactivity, endocrine dysregulation, neuromuscular impairment, and mitochondrial dysfunction [9]. Its prevalence varies widely (3–24%) [10,11] depending on population characteristics and diagnostic methods, and it is particularly high in individuals with chronic diseases or in those with excess adiposity (sarcopenic obesity) [12,13]. Given its profound impact on mobility, cardiometabolic health, immunity, and survival, early detection and timely intervention are critical.

Current screening tools also present important limitations. The SARC-F (an acronym for the domains Strength, Assistance in walking, Rise from a chair, Climb stairs, and Falls) questionnaire is widely accessible and highly specific, but its low sensitivity risks missing early or mild cases. Bioelectrical impedance analysis (BIA) provides a feasible and non-invasive measure of muscle quantity, yet accuracy may vary by device type, hydration status, and population characteristics. Functional performance tests—handgrip strength, Timed Up and Go (TUG), six-minute walk test (6MWT), and the sit-to-stand test—are inexpensive and reliable predictors of mobility decline and mortality, but they are rarely implemented within a unified assessment pathway [14]. In everyday clinical practice, sarcopenia and frailty assessments are often fragmented, relying on only one or two measures, potentially underestimating risk in individuals with complex or borderline phenotypes.

Although validated tools such as SARC-F [15] and SARC-CalF [16] exist, their application in resource-limited community settings is constrained by low sensitivity (SARC-F) or by the need for additional anthropometric measurements (SARC-CalF). Community-based screening for sarcopenia and frailty is thus particularly challenging because of these limitations, limited access to instrumental diagnostics, and the need for low-cost, scalable approaches applicable outside specialized clinical environments. Yet early detection in large populations before the onset of overt disability remains a key public health priority.

A sequential screening strategy—initiating with simple self-reported and strength-based tools (such as SARC-F and handgrip strength) and reserving body composition assessment for screen-positive individuals—may address this gap by improving case detection while preserving feasibility and minimizing instrumental burden. Given the multidimensional nature of these syndromes, there is therefore an urgent need for integrated, pragmatic, and scalable screening strategies combining self-reported, anthropometric, functional, and body composition measures. Such multimodal approaches may improve early identification, support timely intervention, and ultimately reduce the burden of disability in aging populations.

Moreover, the growing availability of digital health tools offers an unprecedented opportunity to extend assessment and intervention beyond traditional clinical settings, supporting continuous monitoring, individualized feedback, and long-term adherence to lifestyle-based therapies.

A two-step approach, initiating with simple self-reported and strength-based screening (e.g., SARC-F and handgrip) and reserving body composition assessment for screen-positive individuals, represents a novel paradigm for community-based detection of sarcopenia and frailty. To our knowledge, this sequential strategy has not previously been systematically operationalized and evaluated within an organized population-level screening programme. Unlike many existing single-step questionnaires or laboratory-centered models that require universal instrumental evaluation, this strategy integrates sensitivity-oriented case finding with targeted confirmatory testing, and is therefore designed to enhance feasibility, scalability, and resource efficiency in real-word settings.

In this context, we conducted a structured screening program integrating SARC-F, handgrip strength, BIA-derived appendicular skeletal muscle index (ASMI), and multiple performance tests to characterize the burden of sarcopenia and frailty in a real-world population. This work addresses a major gap in the current literature: the limited availability of pragmatic, standardized pathways combining muscle strength, muscle mass, and mobility in community settings.

The present study therefore pursued two main aims. First, we conducted a cross-sectional analysis to determine the prevalence of probable and confirmed sarcopenia and frailty according to established operational definitions and to evaluate the diagnostic performance of a stepwise integrated screening algorithm. Moreover, because no single internationally accepted standard exists for frailty screening and because the use of BIA is not always feasible in primary care or community settings, there is a compelling need for a simplified yet accurate diagnostic pathway. For this reason, a secondary aim of this study was to validate a fully standardized two-step screening algorithm, starting with functional and strength-based measures and reserving BIA only for screen-positive cases, with the goal of substantially reducing the use of instrumental assessments without compromising diagnostic accuracy.

## 2. Materials and Methods

### 2.1. Study Design and Setting

This cross-sectional observational study was conducted within a structured screening program for sarcopenia and frailty coordinated by the Department of Public Health of the University of Naples Federico II, within the School of Specialization in Sports and Exercise Medicine. The program was carried out by a multidisciplinary team of physicians, residents, and researchers from the University of Naples Federico II, with the co-participation of academic faculty from the University of Salerno. All procedures followed standardized operating protocols under senior faculty supervision.

### 2.2. Participants and Eligibility Criteria

Consecutive community-dwelling adults aged ≥65 years who voluntarily attended an open public health screening programme for sarcopenia and frailty in September 2025 were considered for inclusion. The programme was advertised through local community channels (poster, flyers, word-of-mouth, and information provided by primary care physicians), allowing self-referral. This recruitment strategy represents a convenience sample of volunteers participating in a public health screening initiative.

**Figure 1:**
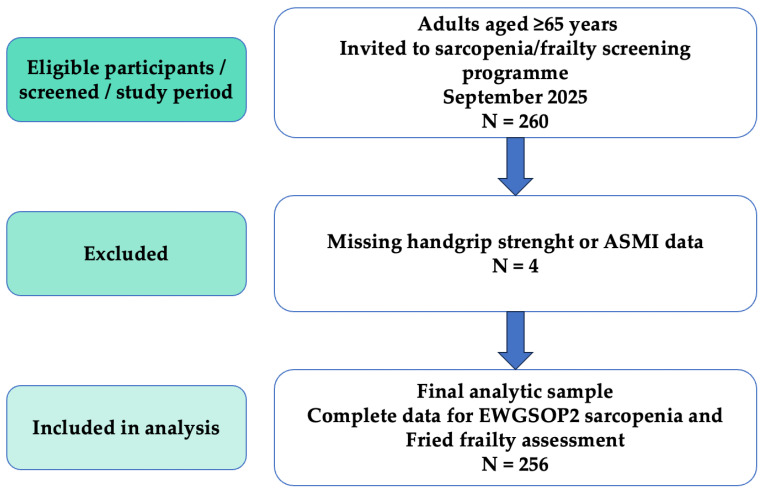
Participant flowchart according to STROBE recommendations. A total of 260 consecutive community-dwelling adults were screened for eligibility in September 2025. Four participants were excluded due to missing handgrip strength or appendicular skeletal muscle index data, yielding a final analytic sample of 256 participants with complete data for sarcopenia and frailty assessment.

Participants were enrolled irrespective of comorbidities or medication use in order to preserve the pragmatic and population-representative nature of the programme. Exclusion criteria were restricted to inability to perform handgrip strength testing or bioelectrical impedance analysis (e.g., implanted electronic devices, limb amputation, or acute illness preventing measurement, such as acute infection, recent hospitalization, or decompensated chronic conditions). The selection process is shown in Figure 1.

### 2.3. Sample Size Calculation

The minimum sample size required to estimate the prevalence of confirmed sarcopenia was calculated using the formula for a single proportion in an infinite population:

(1) $$n=\frac{Z^{2}P\;(1-P)}{d^{2}}$$

where *Z* = 1.96 (95% confidence level), *P* = expected prevalence, and *d* = margin of error. Based on recent Italian and European studies of community-dwelling older adults using EWGSOP2 criteria, an expected prevalence of confirmed sarcopenia of 12% was assumed [10,11,17]. A margin of error of ±4% was selected to balance statistical precision with feasibility in the context of a pragmatic, single-center screening study, and consistent with margins used in comparable geriatric prevalence studies. With a desired precision of ±4%, the calculation yielded a minimum required sample size of 249 participants. The final analytic sample of 256 participants exceeded this requirement, providing a precision of approximately ±3.9%.

### 2.4. Data Collection and Quality Assurance

To ensure maximum standardization, all measurements were performed during two consecutive full-day screening sessions in September 2025. Participants were assessed in the morning after an overnight fast, or after a light meal when fasting was not feasible, and were instructed to refrain from strenuous physical activity, caffeine, and alcohol during the preceding 24 h.

All operators received standardized training in anthropometry, dynamometry, and functional testing prior to the screening. Equipment was calibrated according to manufacturers’ recommendations. Demographic characteristics, medical history, medication use, lifestyle factors (smoking status, alcohol consumption, diet, weekly minutes of physical activity), unintentional weight loss, and self-reported fatigue were recorded using structured case report forms.

All measurements were performed by four trained operators (two physicians and two residents) under the direct supervision of senior faculty. Inter-rater reliability was assessed during the training phase in a pilot sample of 10 participants, yielding intraclass correlation coefficients greater than 0.90 for handgrip strength and all functional performance tests.

### 2.5. Measurements and Operational Definitions

Anthropometry and body composition

- Height (cm) and weight (kg) were measured using a calibrated stadiometer and scale (Gima S.p.A., Gessate, Italy). Body mass index (BMI) was calculated as weight divided by height squared (kg/m^2^).
- Body composition was assessed using a single multi-frequency, eight-electrode BIA device (Biody Xpert ZM, Aminogram SAS, La Ciotat, France) in order to avoid inter-device variability.

Measurements included appendicular skeletal muscle mass (ASMM, kg), fat mass percentage, fat-free mass, and total body water.

- Appendicular skeletal muscle index (ASMI) was calculated as ASMM divided by height squared (kg/m^2^). Low muscle mass was defined according to the EWGSOP2 cut-off values (<7.0 kg/m^2^ for men and <5.5 kg/m^2^ for women).

Muscle Strength

- Handgrip strength was measured using a calibrated digital dynamometer (Gripwise, Matosinhos, Portugal). Three trials were performed for each hand in the standardized seated position (elbow flexed at 90° and forearm in neutral position), and the highest value obtained from the dominant hand was retained for analysis. Low muscle strength was defined according to the EWGSOP2 thresholds (<27 kg in men; <16 kg in women) [3].

Physical performance

- TUG test: Time (in seconds) required to rise from a chair, walk 3 m, return, and sit down again. Impaired performance was defined as >12 s [18].
- 6MWT: Total distance (in meters) walked over 6 min on a flat indoor corridor. Reduced performance was defined as <400 m [19].
- 30 s sit-to-stand test: Number of full stands achieved within 30 s; this measure was used descriptively.

SARC-F Questionnaire and Frailty Assessment

- The validated Italian version of the SARC-F questionnaire [20] was administered according to the original scoring system, where each of the five items scored from 0 to 2, yielding a total score ranging from 0 to 10. A score ≥ 4 was used to indicate increased risk of sarcopenia.
- Frailty was assessed using the Fried frailty phenotype, which includes five criteria: unintentional weight loss, self-reported exhaustion, low physical activity, weakness (handgrip strength), and slowness (gait speed). Participants were classified as: frail (≥3 criteria), prefrail (1–2 criteria), or robust (0 criteria) [5].

Sarcopenia Definitions (EWGSOP2)

- Probable sarcopenia: low muscle strength alone (handgrip strength < 27 kg in men or <16 kg in women) [3].
- Confirmed sarcopenia: probable sarcopenia plus low muscle mass (ASMI < 7 kg/m^2^ in men or <5.5 kg/m^2^ in women).
- Severe sarcopenia: confirmed sarcopenia plus impaired physical performance (TUG > 12 s or 6MWT < 400 m) [3].

Two-Step Screening Algorithm (Index Test)

A pragmatic two-step diagnostic algorithm was evaluated:

1. Step 1 (screening): SARC-F score ≥ 4.
2. Step 2 (confirmation): only in SARC-F-positive participants were handgrip strength and BIA were performed. Confirmed sarcopenia was diagnosed when both low handgrip strength and low ASMI were present.

Diagnostic accuracy of this two-step pathway was compared with the complete EWGOP2 diagnostic flow-chart applied to the entire cohort (reference standard). The sequential (two-step) design was intentional to enable a pragmatic and resource-efficient application in community settings, reserving confirmatory measurements for screen-positive participants only.

### 2.6. Statistical Analysis

Statistical analyses were performed using Stata/SE 16.1 (StataCorp LLC, College Station, TX, USA). The distribution of continuous variables was assessed using the Shapiro–Wilk test. Normally distributed variables are reported as mean ± standard deviation (SD), while non-normally distributed variables are presented as median and interquartile range (IQR). Categorical variables were expressed as absolute numbers and percentages.

Prevalence estimates of probable, confirmed, and severe sarcopenia (according to the 2019 EWGSOP2 criteria) and of pre-frailty/frailty (Fried phenotype) were calculated with 95% confidence intervals (CIs) using the Wilson score method.

Between-group comparisons were performed using Student’s *t*-test or the Mann–Whitney U test for continuous variables, and χ^2^ test or Fisher’s exact test for categorical variables, as appropriate. Associations between ASMI, handgrip strength, and physical performance measures were assessed using Pearson’s or Spearman’s correlation coefficients according to data distribution.

Diagnostic accuracy of the pragmatic two-step screening algorithm was evaluated against the full EWGSOP2 diagnostic flow-chart, which served as the reference standard. Sensitivity, specificity, positive and negative predictive values, and positive and negative likelihood ratios were calculated with 95% CIs using the Wilson score method. The area under the receiver operating characteristic (ROC) curve (AUC) and its 95% CI were estimated non-parametrically with DeLong’s method. The proportion of bioelectrical impedance analyses spared by the two-step strategy was reported with exact binomial 95% CIs.

Given the limited number of confirmed sarcopenia cases (*n* = 37), multivariable logistic regression adhered to the “one predictor per 10 events” rule to minimize risk of overfitting. The model included four a priori selected clinically relevant variables: age, low BMI (<22 km/m^2^), physical inactivity (<150 min/week), and SARC-F score. Multicollinearity was excluded (all variance inflation factors < 2.0). A confirmatory backward elimination procedure (removal criterion *p* > 0.10) was applied to the pre-specified variables. Internal validation of diagnostic accuracy was performed using 1000 bootstrap resamples; the optimism-corrected c-statistic differed by <0.02 from the apparent c-statistic. Results were reported as adjusted odds ratios (aORs) with 95% CIs. Model significance, calibration, and discrimination were assessed using the likelihood-ratio test, the Hosmer–Lemeshow goodness-of-fit test, and c-statistic, respectively.

Sensitivity analyses were conducted using total body water percentage (%TBW = total body water/body weight × 100) as a proxy for hydration status, excluding participants with %TBW > 60%. Additional analyses excluded participants receiving diuretic therapy.

Agreement between confirmed sarcopenia (EWGSOP2) and frailty (Fried phenotype) was quantified using Cohen’s κ coefficient with 95% CI.

All statistical tests were two-sided, and statistical significance was set at *p* < 0.05. The study was reported in accordance with the STROBE guidelines for observational studies and the STARD 2015 recommendations for diagnostic accuracy research.

### 2.7. Ethical Considerations

The study was conducted in accordance with the principles of the Declaration of Helsinki. All collected data were treated as strictly confidential and used exclusively for the purposes of the community-based screening initiative, in compliance with Legislative Decree 196/2003 and EU Regulation 2016/679 (General Data Protection Regulation, GDPR). Personal identifiers were not included in the analytic dataset, and all identifiable raw data were collected and temporarily stored by FDM, who acted as the data controller in accordance with Articles 28 and 39 of the GDPR.

Given the non-interventional, observational nature of the initiative—involving retrospective analysis of anonymized data collected during a voluntary public health screening programme with written informed consent from participants—formal ethics committee approval was not required under Italian legislation for this type of low-risk public health and quality improvement activity. This is consistent with the exclusion of non-interventional studies from the scope of regulations governing interventional clinical trials (as defined in Legislative Decree No. 211/2003, which implements Directive 2001/20/EC and explicitly delimits its application to interventional trials). Participants were fully informed about the voluntary nature of participation and provided written informed consent prior to enrolment, ensuring compliance with data protection requirements. All identifiable data will be permanently destroyed once aggregated analyses have been completed.

## 3. Results

A total of 260 consecutive community-dwelling adults aged ≥65 years underwent screening for sarcopenia and frailty within a structured program coordinated by the School of Specialization in Sports and Exercise Medicine (Department of Public Health of the University of Naples Federico II), with academic collaboration from the University of Salerno. To ensure maximal standardization, all clinical, functional, and instrumental assessments were performed during two consecutive full-day sessions in September 2025. Four participants were excluded due to missing handgrip strength and/or appendicular skeletal muscle index data, resulting in a final analytic sample of 256 participants (Figure 1).

Baseline characteristics are summarized in Table 1. The mean age was 74.6 ± 6.8 years (range 65–79 years), and women represented 45.3% of the cohort (*n* = 116). The mean BMI was 27.8 ± 4.9 kg/m^2^, with 38 participants (14.8%) presenting a BMI < 22 kg/m^2^, a recognized high-risk category for sarcopenia in European older adults. A total of 142 participants (55.5%) reported physical activity < 150 min/week of moderate intensity. The mean SARC-F score was 2.3 ± 2.1; 59 individuals (23.0%) scored ≥4, indicating increased risk of sarcopenia.

Prevalence estimates for sarcopenia and frailty are reported in Table 2. Probable sarcopenia (low muscle strength alone, EWGSOP2) was present in 74 participants (28.9%, 95% CI 23.7–34.7%). Confirmed sarcopenia was diagnosed in 37 participants (14.5%, 95% CI 10.7–19.1%). Of these, 23 (62.2% of confirmed cases; 9.0% of the total sample, 95% CI 6.0–13.2%) had severe sarcopenia.

According to the Fried phenotype, 98 participants (38.3%) were prefrail and 31 (12.1%, 95% CI 8.6–16.7%) were frail.

**Figure 2:**
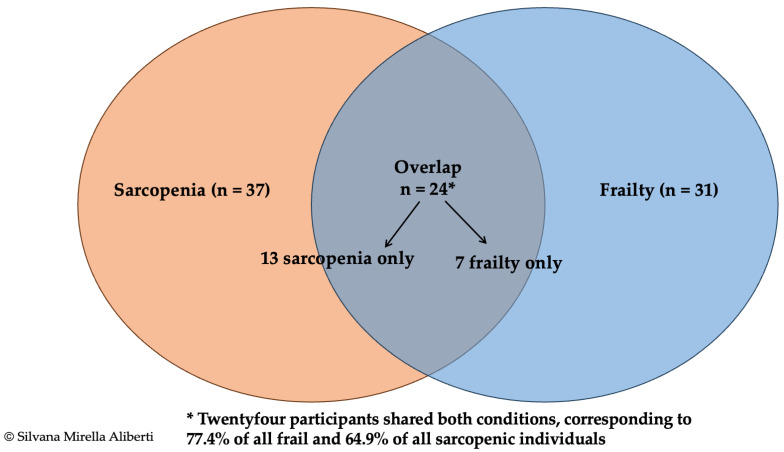
Venn diagram illustrating the overlap between confirmed sarcopenia (EWGSOP2 criteria [3], *n* = 37) and physical frailty (Fried phenotype [5], *n* = 31) in 256 community-dwelling older adults. The diagram shows 13 participants with sarcopenia only, 7 with frailty only, and 24 presenting both conditions. The overlap represents 77.4% of all frail participants and 64.9% of those with confirmed sarcopenia. Agreement between classifications was moderate to substantial (Cohen’s κ = 0.62; 95% CI 0.49–0.74).

Substantial overlap was observed: 24 of the 31 frail participants (77.4%) had confirmed sarcopenia, and 26 of the 37 sarcopenic participants (70.3%) were prefrail or frail. Agreement between the two classifications was moderate-to-substantial (Cohen’s κ = 0.62, 95% CI 0.49–0.74; *p* < 0.001). The distribution of exclusive and overlapping cases is illustrated in Figure 2.

**Figure 3:**
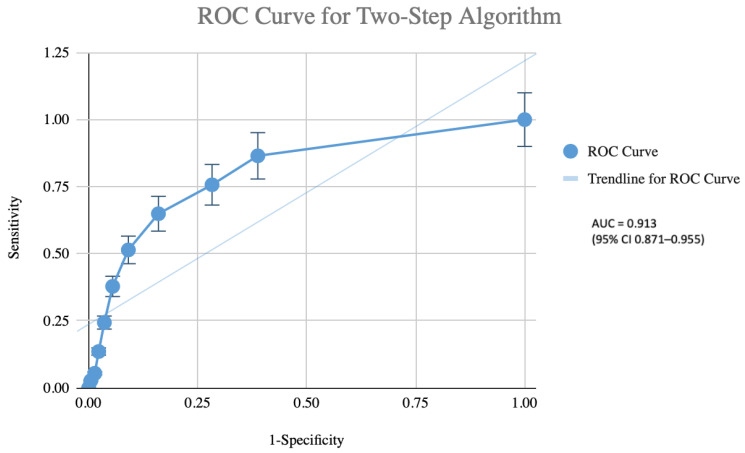
Receiver operating characteristic (ROC) curve of the pragmatic two-step screening algorithm (Step 1: SARC-F score ≥ 4; Step 2: handgrip strength measurement and bioelectrical impedance analysis performed only in screen-positive participants) for the identification of confirmed sarcopenia (EWGSPO2 criteria) in 256 community-dwelling older adults. The area under the ROC curve was 0.913 (95% CI 0.871–0.955), indicating excellent diagnostic accuracy. The dotted diagonal line represents the line of no discrimination (AUC = 0.5).

Diagnostic performance of the pragmatic two-step algorithm is presented in Table 3 and Figure 3. Using the full EWGSOP2 diagnostic flowchart applied to the entire cohort as the reference standard, the algorithm (Step 1: SARC-F score ≥ 4; Step 2: handgrip strength + BIA only in Step 1 positive individuals) correctly identified 34 of the 37 confirmed sarcopenia cases. Sensitivity was 91.9% (95% CI 78.1–98.3%) and specificity of 81.3% (95% CI 75.7–86.0%). The positive predictive value was 47.2%, and the negative predictive value was 98.2% (95% CI 95.0–99.5%). The positive and negative likelihood ratios were 4.91 and 0.10, respectively. Implementation of this algorithm would have obviated BIA testing in 138 participants (53.9% of the cohort). The AUC was 0.913 (95% CI 0.871–0.955), indicating excellent discriminatory ability. Bootstrap internal validation (1000 resamples) yielded an optimism-corrected AUC of 0.911, confirming minimal optimism bias.

Sensitivity analyses excluding participants with elevated total body water (%TBW > 60%, *n* = 28; final *n* = 228) yielded comparable diagnostic performance (sensitivity 93.9%, specificity 82.6%, AUC 0.918). Similar results were obtained when excluding participants using diuretics (*n* = 24; final *n* = 232), with sensitivity of 94.1%, specificity of 77.8%, and an AUC of 0.915, confirming the robustness of the main findings to potential hydration-related bias.

Multivariable logistic regression (Table 4) identified four factors independently associated with confirmed sarcopenia: older age (aOR 1.08 per year, 95% CI 1.03–1.13; *p* = 0.001), BMI < 22 kg/m^2^, which showed the strongest association (aOR 4.38, 95% CI 2.12–9.04; *p* < 0.001), physical inactivity (aOR 3.67, 95% CI 1.71–7.88; *p* = 0.001), and SARC-F score (aOR 1.94, 95% CI 1.55–2.43; *p* < 0.001). Female sex was not retained in the final model. The model showed good calibration (Hosmer–Lemeshow χ^2^ = 6.81, df = 8, *p* = 0.71) and excellent discrimination (c-statistic = 0.89).

Prevalence and overlap estimates are unadjusted descriptive statistics. Associations with potential confounders (age, sex, BMI) were explored in multivariable analysis (Table 4) and stratified baseline characteristics (Table 1).

## 4. Discussion

The present study provides novel and practice-oriented evidence on the relationship between sarcopenia and physical frailty within a population-based screening programme conducted in a real-world public health setting. Unlike most previous investigations conducted in specialized geriatric or outpatient contexts, this study operationalized a fully standardized diagnostic pathway within an organized community screening infrastructure, generating implementation-level data with direct relevance for public health practice. Our findings confirm that sarcopenia and frailty frequently coexist and share substantial conceptual and clinical overlap, while also demonstrating the feasibility of integrating combined screening into routine population-based workflows.

Since the seminal work of Rosenberg [21], which framed sarcopenia as a central biological manifestation of aging, skeletal muscle health has become a cornerstone in the understanding of vulnerability in later life. In parallel, the evolution of the frailty construct– shaped by early contributions of Guralnik and colleagues [22]—has progressively shifted geriatric research towards prevention and functional preservation. Although sarcopenia and frailty were initially conceptualized within distinct disciplinary traditions, their convergence is now widely recognized [23,24]. This convergence, described by Bauer and Sieber [25] and critically discussed by the group of Cesari [26], challenges simplistic causal models and supports a unified framework centered on declining physical function as a shared biological substrate.

This conceptual framework has been reinforced by work from Ferrucci [27], Theou [28], and Abellan van Kan [29], who consistently position mobility limitation and reduced physiological reserve as lying at the core of both syndromes. In this context, our findings contribute original operational evidence by quantifying the degree of overlap between EWGSOP2-defined sarcopenia and the Fried frailty phenotype within a population-based screening framework, and by demonstrating moderate-to-substantial agreement between these classifications. Our prevalence estimates for confirmed sarcopenia (14.5%) and frailty (12.1%) are consistent with recent meta-analyses in European community-dwelling older adults using EWGSOP2 criteria (pooled estimates approximately 11–23%) and the Fried phenotype (around 18%) [11,30,31]. Importantly, by embedding this evaluation into an organized screening programme, the present study extends existing knowledge from descriptive epidemiology toward practical implementation.

From a preventive medicine perspective, this overlap between sarcopenia and frailty has major implications. According to the classical framework of primary, secondary, and tertiary prevention [32], the coexistence of these conditions in the presence of established disability often represents a late and less reversible stage, resembling the clinical trajectory of cachexia described by Rolland [33]. In contrast, the frequent identification of impaired physical function in the absence of overt disability in our cohort represents a quintessential window for secondary prevention. Earlier work by Subra [34] and Maggio [35] highlighted that this preclinical phase offers the greatest potential for modifying trajectories of decline. Our study provides empirical support for this model by showing that a simplified, two-step diagnostic algorithm can reliably identify confirmed sarcopenia at this critical stage within a community-based screening context.

Recent literature has increasingly emphasized the value of simple, performance-based measures for early risk stratification. Reviews by Dodds et al. [4] and contemporary mechanistic syntheses by Ye and colleagues [1] have reinforced the biological and clinical rationale for integrated approaches to frailty and sarcopenia. In contrast to predominantly conceptual or laboratory-based work, the present study translates these insights into a reproducible, scalable and resource-efficient screening pathway tested at the population level, thereby addressing a key gap between theory and operational practice.

The pragmatic two-step algorithm implemented in this study—SARC-F followed by handgrip strength and selective BIA—demonstrated excellent diagnostic performance, including high discrimination and strong rule-out capability, while substantially reducing the need for instrumental body composition assessment. Although offering a feasible and scalable option for community screening, the algorithm is not intended as a definitive public health solution and requires validation in larger, multicenter cohorts to confirm its performance across diverse populations. Compared with SARC-F alone, which shows high specificity but limited sensitivity, or SARC-CaIF, which incorporates calf circumference but requires additional measurement, our approach combines questionnaire screening with objective strength assessment prior to selective BIA, thereby improving sensitivity and feasibility in community settings while minimizing instrumental burden.

The relatively low positive predictive value is expected in a screening context with moderate prevalence of confirmed sarcopenia, in which the algorithm deliberately prioritizes sensitivity and negative predictive value to minimize missed cases, consistent with the objective of rule-out screening in community populations. The hierarchical and sequential structure of the algorithm thus reflects a conscious design choice to facilitate scalable implementation in settings with limited access to instrumental tools, in line with calls for simplified case-finding pathways. Against a background of persistent heterogeneity in operational definitions and cut-offs, as documented by EWGSOP2 [3] and the NIH Foundation initiatives [36,37,38,39], these findings provide concrete evidence that diagnostic simplification can be achieved without compromising accuracy.

The independent associations observed between confirmed sarcopenia and older age, low BMI, physical inactivity, and higher SARC-F scores support the interpretation of sarcopenia as the product of both biological ageing and modifiable lifestyle factors. In particular, low BMI and sedentariness represent actionable targets for preventive interventions centered on resistance exercise and nutritional optimization, consistent with evidence linking mobility performance measures to adverse ageing outcomes [40,41,42,43]. This positions the proposed screening pathway not merely as a diagnostic instrument, but as a practical entry point for structured preventive care.

From a broader public health perspective, the simplicity and low resource requirements of the algorithm facilitate its integration into routine primary care or community health checks, enabling large-scale early identification of at-risk older adults and supporting population-level strategies to reduce disability and healthcare burden.

More broadly, healthy ageing research increasingly recognizes that functional decline emerges from the interaction of biological, environmental and social determinants. Large population-based studies demonstrate that habitual physical activity, dietary patterns, social connectedness, and environmental exposures substantially shape resilience in later life [40,43,44], supporting an integrated view of muscle health as a central mediator of healthspan. Within this perspective, sarcopenia and frailty are best understood not as isolated entities but as interconnected manifestations of diminishing physiological reserve, with physical function representing the key measurable axis through which vulnerability can be identified and potentially modified.

The heterogeneity of aging observed in our cohort is consistent with emerging evidence describing distinct phenotypic profiles across older populations, including variability in clinical burden, nutritional habits, psychological well-being and community-level determinants of health [44]. These observations reinforce the notion that protective behaviors, such as sustained physical activity, adherence to specific dietary patterns, and robust social engagement, may contribute to functional resilience and longevity. Incorporating such multidimensional determinants into sarcopenia and frailty prevention frameworks may enhance early risk stratification and guide more personalized intervention strategies.

Conceptual work increasingly supports prioritizing physical function as a unifying framework for integrating sarcopenia and frailty into research and clinical practice [26,45,46,47]. This integrative, function-centered vision is further reinforced by recent models identifying physical function as a measurable hallmark of ageing [48,49,50]. Our findings align with this perspective: the strong diagnostic performance of a simple, function-centered algorithm supports the view that muscle strength and performance represent modifiable hallmarks of ageing capable of directing preventive strategies at both individual and population levels.

Emerging insights from exposome research further strengthen this interpretation. Environmental exposures, microbiome diversity, lifestyle behavior and psychosocial resilience jointly shape ageing trajectories and contribute to longevity, particularly in regions characterized by sustained physical activity, Mediterranean dietary patterns rich in polyphenols and probiotics, strong social cohesion, and biodiverse natural environments [51]. These factors converge on the preservation of muscle function and attenuate metabolic and inflammatory decline—mechanisms closely linked to both sarcopenia and frailty. This reinforces the public health relevance of early, function-based detection and supports the role of screening as an operational bridge between preventive theory and practice.

The strengths of this study include a rigorously standardized assessment protocol, the integration of validated diagnostic frameworks (EWGSOP2 and Fried phenotype), and the evaluation of a pragmatic two-step algorithm within a real-world community screening workflow.

Several limitations should nevertheless be acknowledged. The cross-sectional design precludes causal inference and does not permit assessment of prognostic validity or longitudinal outcomes. Participants were consecutively enrolled from a voluntary community screening programme, representing a convenience sample that may limit generalizability. The study was conducted in a single Italian region with participants predominantly of European ancestry, potentially restricting applicability to populations with different ethnic backgrounds, dietary patterns, or healthcare systems. The single-center design, moderate sample size, and absence of external validation further limit generalizability. Although measurements were standardized and performed using a single BIA device under controlled conditions (morning assessment after fasting/light meal and abstinence from exercise/caffeine/alcohol), BIA-derived ASMI may be influenced by hydration status or diuretic use. While these factors were not directly measured, sensitivity analyses using total body water as a proxy and excluding diuretic users confirmed the robustness of the findings. Automated variable selection may introduce a risk of overfitting despite adherence to recommended event-to-predictor ratios and low multicollinearity; penalized regression approaches could be considered in future studies with larger samples. The Fried phenotype does not capture cognitive, psychological, or social domains of frailty, and no alternative body composition method (e.g., DXA) was available for comparison. Missing data were minimal and handled via complete case analysis.

Potential barriers to implementation include the need for basic training in handgrip dynamometry, the time required for screening (approximately 10–15 min per participant), minimal equipment costs, and variability in acceptability outside voluntary settings. Future longitudinal and multicenter studies are needed to assess prognostic value, external validity, and whether early identification translates into improved outcomes with targeted interventions.

## 5. Conclusions

In this large population-based screening initiative, we demonstrated that a pragmatic, function-centered approach can reliably identify older adults with sarcopenia and physical frailty in a real-world public health setting. The strong concordance between the two conditions reinforces the notion that they represent interconnected manifestations of declining physiological reserve, primarily rooted in neuromuscular impairment and reduced physiological reserve. Our simplified two-step algorithm—SARC-F combined with handgrip strength, followed by selective BIA—showed excellent diagnostic performance and is feasible for large-scale implementation, supporting current recommendations to prioritize physical function as a core metric of aging and providing an actionable pathway for secondary prevention, enabling early identification of at-risk individuals before disability emerges.

Independent predictors of confirmed sarcopenia—older age, low BMI, physical inactivity, and higher SARC-F score—highlight the joint influence of biological and lifestyle factors and identify clear targets for preventive interventions. Integrating structured exercise, nutritional optimization, and behavioral support within community programs may therefore enhance the capacity of public health systems to preserve functional independence and extend healthspan.

Although the cross-sectional design precludes causal inference and generalizability is limited by the single-region setting, the standardized methodology and operational framework strengthen the applied relevance of our findings. Importantly, the algorithm’s ability to identify functional vulnerability before disability emerges aligns closely with the principles of modern preventive medicine, which emphasize early detection of modifiable risk factors to interrupt trajectories of decline before irreversible disability occurs. Future research should prioritize pilot implementation of this algorithm in diverse community and primary care settings, as well as longitudinal validation in larger, multicenter cohorts to determine whether early identification, combined with targeted interventions (e.g., resistance training and nutritional optimization), can improve long-term functional and clinical outcomes. Overall, these findings offer meaningful support for a unified, function-oriented model of aging and provide a scalable framework for implementing preventive strategies within community settings.

## Figures and Tables

**Table 1:** Baseline characteristics of the study population (*n* = 256).

Characteristic	Overall (*n* = 256)	Male (*n* = 140)	Female (*n* = 116)
Age, years (mean ± SD)	74.6 ± 6.8	75.1 ± 6.9	74.3 ± 6.7
Age range, years	65–79	65–79	65–79
Female sex, n (%)	116 (45.3)	-	-
Body mass index (BMI), kg/m^2^ (mean ± SD)	27.8 ± 4.9	27.5 ± 4.2	28.0 ± 5.3
BMI < 22 kg/m^2^, n (%)	38 (14.8)	20 (14.3)	18 (15.5)
Hypertension, n (%)	178 (69.5)	98 (70.0)	80 (69.0)
Type 2 diabetes, n (%)	52 (20.3)	30 (21.4)	22 (19.0)
Dyslipidemia, n (%)	112 (43.8)	62 (44.3)	50 (43.1)
History of cancer, n (%)	29 (11.3)	18 (12.9)	11 (9.5)
Cardiovascular disease, n (%)	71 (27.7)	42 (30.0	29 (25.0)
Chronic respiratory disease, n (%)	31 (12.1)	19 (13.6)	12 (10.3)
Number of chronic medications, median (IQR)	4 (2–6)	4 (3–6)	4 (2–6)
Physical activity < 150 min/week, n (%)	142 (55.5)	78 (55.7)	64 (55.2)
Current or former smoker, n (%)	88 (34.4)	62 (44.3)	26 (22.4)
SARC-F score (mean ± SD)	2.3 ± 2.1	1.9 ± 1.8	2.6 ± 2.2
SARC-F ≥ 4, n (%)	59 (23.0)	32 (22.9)	27 (23.3)

Note: SD = standard deviation; IQR = interquartile range. BMI < 22 kg/m^2^ represents the EWGSOP2 high-risk category. Hypertension, diabetes, dyslipidemia, and cancer history refer to personal medical history. Physical activity was self-reported in minutes/week of moderate intensity.

**Table 2:** Prevalence, Overlap, and Agreement between Confirmed Sarcopenia and Physical Frailty (*n* = 256).

**A.** **Prevalence of sarcopenia and frailty**						
Condition			n (%)		95% CI	
Probable sarcopenia			74 (28.9%)		23.7–34.7	
Confirmed sarcopenia			37 (14.5%)		10.7–19.1	
Severe sarcopenia			23 (9.0%)		6.0–13.2	
Prefrailty (Fried)			98 (38.3%)		32.3–44.7	
Frailty (Fried)			31 (12.1%)		8.6–16.7	
**B.** **Overlap Indicators (analytical)**						
Relationship			Value		95% CI	*p*-value
Frail individuals with confirmed sarcopenia			24/31 (77.4%)		60.2–88.6	<0.001
Confirmed sarcopenia cases that were prefrail or frail			26/37 (70.3%)		53.0–83.2	<0.001
**C.** **Agreement between EWGSOP2 confirmed sarcopenia and Fried frailty phenotype**						
Metric	Value	95% CI		*p*-value		
Cohen’s κ	0.62	0.49–0.74		<0.001		

Note: 95% confidence intervals were calculated using the Wilson score method. Cut-offs according to EWGSOP2 2019 consensus [3] and Fried phenotype [5]. Overlap and agreement between confirmed sarcopenia and frailty are illustrated in Figure 2.

**Table 3:** Diagnostic performance of two-step screening algorithm for confirmed sarcopenia versus the full EWGSOP2 criteria (*n* = 256).

Metric	Value	95% CI
Sensitivity	91.9%	78.1–98.3
Specificity	81.3%	75.7–86.0
Positive predictive value (PPV)	45.3%	35.3–59.5
Negative predictive value (NPV)	98.2%	95.0–99.5
Positive likelihood ratio (LR+)	4.91	3.58–6.73
Negative likelihood ratio (LR−)	0.10	0.03–0.29
Area under the ROC curve (AUC)	0.913	0.871–0.955
BIA measurement spared	138/256 (53.9%)	47.8–60.0

Note: 95% confidence intervals for proportions were calculated using the Wilson method, for likelihood ratios using the log method, and for the AUC using DeLong’s method. The two-step algorithm consisted of SARC-F ≥ 4 as the screening step, followed by handgrip strength and BIA only in screen-positive participants. Sensitivity analyses excluding participants with elevated hydration (%TBW > 60%) or diuretic use yielded comparable performance (AUC 0.915–0.918; see text). The reference standard was the full EWGSOP2 diagnostic criteria [3]. Cut-offs for low muscle strength and ASMI were defined according to EWGSOP2 [3].

**Table 4:** Multivariable logistic regression analysis for confirmed sarcopenia.

Dependent Variable: Confirmed Sarcopenia (Yea/No) *N* = 256 Participants; 37 Cases			
Independent Variable	Adjusted OR	95% CI	*p*-Value
Age	1.08	1.03–1.13	<0.01
BMI < 22 kg/m^2^	4.38	2.12–9.04	<0.001
Physical inactivity (<150 min/week)	3.67	1.71–7.88	<0.01
SARC-F score (per point)	1.94	1.55–2.43	<0.001

Note: OR—odds ratio; 95% CI—Confidence Interval. Model fit: Log likelihood = −89.42; likelihood-ratio χ^2^ = 78.51 (4 df), *p* < 0.001; Hosmer-Lemeshow χ^2^ = 6.81, *p* = 0.71; c-statistic = 0.89.

## Data Availability

The raw data supporting the conclusions of this article will be made available by the corresponding author on reasonable request, due to privacy restrictions related to personal health information protected by EU General Data Protection Regulation (GDPR).

## Abbreviations

The following abbreviations are used in this manuscript:

6MWT	Six-minute Walk Test
aOR	Adjusted Odds Ratio
ASMI	Appendicular Skeletal Muscle Index
ASMM	Appendicular Skeletal Muscle Mass
AUC	Area Under the Curve
BIA	Bioelectrical Impedance Analysis
BMI	Body Mass Index
EWGSOP2	European Working Group on Sarcopenia in Older People
GDPR	General Data Protection Regulation
IQR	Interquartile Range
ROC	Receiver Operating Characteristic
SARC-F	Strength, Assistance in walking, Rise from a chair, Climb stairs, Falls
SARC-CalF	SARC-F Combined With Calf Circumference
TUG	Timed Up and Go
